# Single-port endoscopic-sentinel lymph node biopsy combined with indocyanine green and carbon nanoparticles in breast cancer

**DOI:** 10.1007/s00464-023-10018-9

**Published:** 2023-07-17

**Authors:** Zi-Han Wang, Tian-Ran Gang, Shan-Shan Wu, Can Lu, Guo-Xuan Gao, Wei Xu, Guo-Qian Ding, Xiang Qu, Zhong-Tao Zhang

**Affiliations:** 1 Department of Breast Disease, Peking University People’s Hospital, Beijing, 100044 China; 2 Department of Anesthesiology, China-Japan Friendship Hospital, Beijing, 100029 China; 3 Department of Clinical Epidemiology and Evidence-Based Medicine, Beijing Friendship Hospital, 95 Yong-an Road, Xi-Cheng District, Beijing, 100050 China; 4 Department of Breast Surgery, Beijing Daxing District Maternal and Child Health Hospital, Beijing, China; 5 Department of General Surgery, Beijing Friendship Hospital, Capital Medical University, 95 Yong-an Road, Xi-Cheng District, Beijing, 100050 China

**Keywords:** Breast cancer, Endoscopic, Single-port, Indocyanine green, Sentinel lymph node biopsy

## Abstract

**Background:**

In order to explore the surgical safety and the reliability of axillary staging of single-port endoscopic-sentinel lymph node biopsy, we combined it with indocyanine green that was excited by near-infrared fluorescence endoscopy and carbon nanoparticles as a tracer and compared this method to conventional open sentinel lymph node biopsy.

**Methods:**

This is a retrospective and observational study, there were 20 patients in each group and the total sample size was 60: Group 1, single-port endoscopic-sentinel lymph node biopsy combined with indocyanine green and carbon nanoparticles; Group 2, single-port endoscopic-sentinel lymph node biopsy with carbon nanoparticles only; Group 3, conventional sentinel lymph node biopsy with indocyanine green and carbon nanoparticles. Sentinel lymph node detection and upper extremity function were determined to measure the safety and efficacy of the novel single-port endoscopic-sentinel lymph node biopsy (SPE-SLNB) technique to the standard conventional sentinel lymph node biopsy technique.

**Results:**

The detection rate of sentinel lymph nodes was 100% in Group 1, 100% in Group 2, and 95% in Group 3. There were no significant differences in upper arm function and pain scores between the three groups.

**Conclusion:**

The novel technique of combining indocyanine green and carbon nanoparticles with single-port endoscopic-sentinel lymph node biopsy achieved a similar detection rate and mean number of sentinel lymph nodes as conventional sentinel lymph node biopsy. Traditional open surgery requires two different incisions for breast surgery and SLNB. While the most important advantage of SPE-SLNB is that two procedures can be effectively performed through a single-port in the axilla Therefore, for patients who meet the indications, single-port endoscopic-sentinel lymph node biopsy is as safe and reliable as conventional sentinel lymph node biopsy but has the aesthetic advantage of only one incision.

In the late twentieth century, in developing countries, one of the greatest advances in clinical surgery was the gradual maturity of the theory of minimally invasive surgery and then the rapid development of endoscopic surgery to make that theory a reality. Endoscopic technology is precise, minimally invasive, and can protect the function of the surgical area (e.g., upper extremity function is protected during endoscopic lymph node biopsy). As an important female organ, breast surgery has benefitted from the development of minimally invasive surgery because of the cosmetic effect and postoperative quality of life of patients [1–3].

Single-port endoscopic surgery technology has several advantages in breast surgery including being less invasive and using a small, hidden surgical incision, which achieves a beneficial postoperative aesthetic effect. In recent years, the application of single-port endoscopic-assisted technology in the field of breast cancer has become more and more extensive. A number of studies have shown that the cosmetic effect and safety of single-port endoscopic subcutaneous mastectomy, single-port endoscopic breast reconstruction, and single-port endoscopic breast-conserving surgery are equivalent to traditional open surgery [4–6].

Sentinel lymph node biopsy (SLNB) is an important part of breast cancer staging and can avoid axillary lymph node dissection when the sentinel lymph node (SLN) metastases fulfill the Z0011 criteria [7]. Conventional SLNB (C-SLNB) requires an incision to remove the SLNs in addition to the incisions used to perform the breast surgery. If a surgeon is performing single-port endoscopic breast surgery for treatment of breast cancer, then single-port endoscopic SLNB (SPE-SLNB) implemented simultaneously could avoid additional incisions and result in a better aesthetic outcome.

In terms of sensory function, the anatomical location of the sentinel lymph node is close to the initiation point of the intercostal nerve from the chest wall, thus causing the injury of the intercostal nerve in some patients undergoing SLNB [8, 9], causing irreversible numbness, pain or other paresthesia in the inner upper arm or shoulder. According to previous reports, compared with conventional axillary surgery, endoscopic axillary surgery has advantages in operative outcomes, complication reduction, function conservation, and cosmetics [10].

At present, few studies have shown whether SPE-SLNB and C-SLNB have the same reliability in the detection rate and number of SLNs. In order to explore the surgical safety and reliability in axillary staging of SPE-SLNB, our center created a novel technique of combining indocyanine green (ICG) and carbon nanoparticles (CN) as a tracer and utilized an endoscopic fluorescence imaging system through a single-port. We then compared this novel SPE-SLNB method to C-SLNB. We present the following article in accordance with the STARD reporting checklist.

## Materials and methods

### Ethics statement

This study was approved by the Ethics Committee of the Beijing Friendship Hospital, Capital Medical University (2019-P2-058-02). Written informed consent was obtained from all patients before surgery.

### Patients

Procedures were performed on breast cancer patients (*N* = 60) between March 2019 and May 2020 at the Beijing Friendship Hospital. Patients with early invasive breast cancer (stage I and II) as confirmed by core needle biopsy and with clinically negative axilla were enrolled in the present study. Patients with tumors > 5 cm, clinically or radiologically suspicious lymph nodes, inflammatory breast cancer, distant metastatic tumor, previous axillary surgery, or hypersensitivity to iodine or ICG were excluded from the study.

**Fig. 1:**
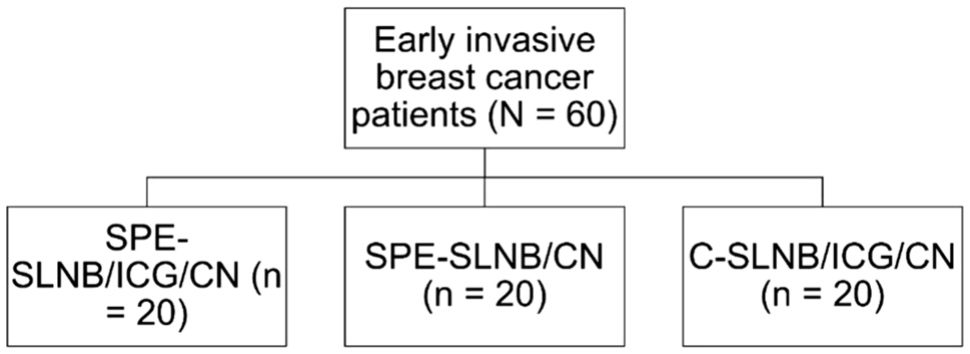
Flow diagram of patient procedure groups. *SPE-SLNB* Single-port endoscopic sentinel lymph node biopsy; *ICG* Indocyanine green; *CN* Carbon nanoparticle; *C-SLNB* Conventional sentinel lymph node biopsy

This is a retrospective and observational study. We review the previous clinical cases, and then fully communicate with patients before surgery to decide which group patients should be assigned to base on the principle of respecting patients' personal wishes. There were 20 patients in each group and the total sample size was 60: (Fig. 1). In Group 1, Twenty patients underwent SPE-SLNB combined with ICG and CNs. In Group 2, Another 20 patients underwent SPE-SLNB using CNs only. Endoscopic breast surgery (including subcutaneous mastectomy, single-port endoscopic breast reconstruction, and single-port endoscopic breast-conserving surgery) was performed on patients in both Group 1 and Group 2 at the same time as SPE-SLNB to avoid a second axillary incision. The remaining 20 patients in Group 3 who accepted open breast surgery underwent C-SLNB using ICG and CNs.

### Surgical techniques

#### Group 1: SPE-SLNB combined with ICG and CNs

The patient was in a supine position with a high shoulder cushion on the affected side. The upper limb was wrapped in a sterile towel and placed at a 90° abduction. ICG (25 mg, Yichuang Pharmaceutical LLC, Dandong, China) was dissolved in 10 mL sterilized distilled water before use, and the mass concentration was 2.5 mg/mL after dissolving. Then the ICG was further diluted to 0.5 mg/mL in 1 mL of sterilized distilled water for use. Intradermal injection of 0.3 mg/mL of ICG and 0.2 mL of CNs (Chongqing LUMMY Pharmaceutical, Chongqing, China) were injected at the outer and lower margins of the areola. Tumescent solution was injected into the axilla to facilitate liposuction. The formula of the tumescent solution was 1 mg adrenaline and 20 mL of 2% lidocaine mixed with 250 mL of 0.9% sodium chloride and 250 mL of sterilized distilled water. A total of 100 mL of tumescent solution was injected into the SLN region with a blunt lipolysis needle at the top. After 15 min, we performed liposuction in this area.

**Fig. 2:**
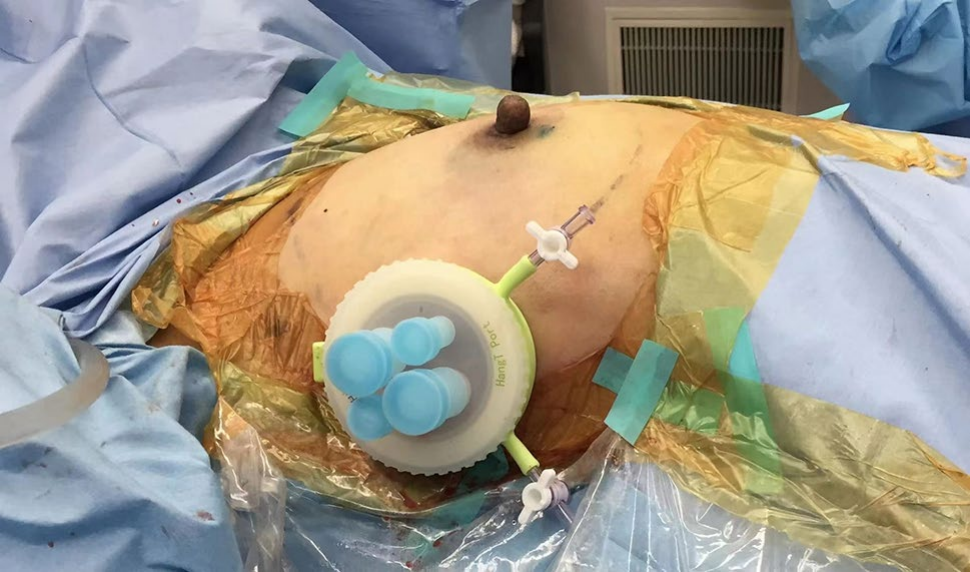
The single-port incision was created with the single-port insufflation kit. Indocyanine green and carbon nanoparticles were injected at the out and lower margins of the areola

**Fig. 3:**
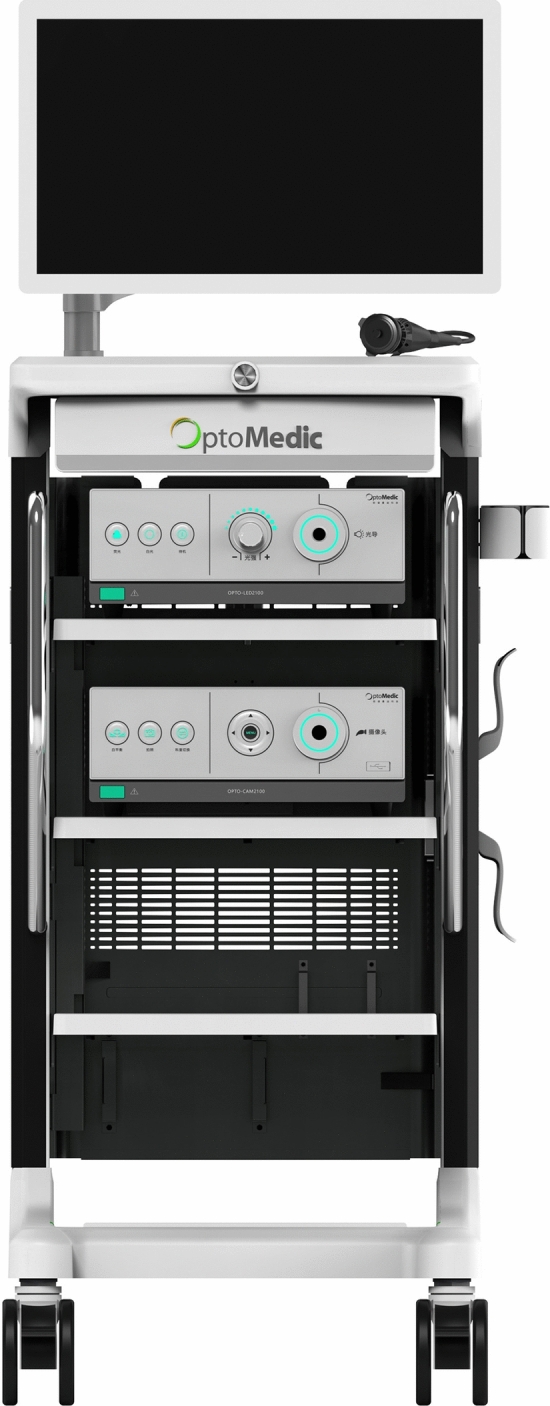
The FloNaviTM Endoscopic Fluorescence Imaging System

**Fig. 4:**
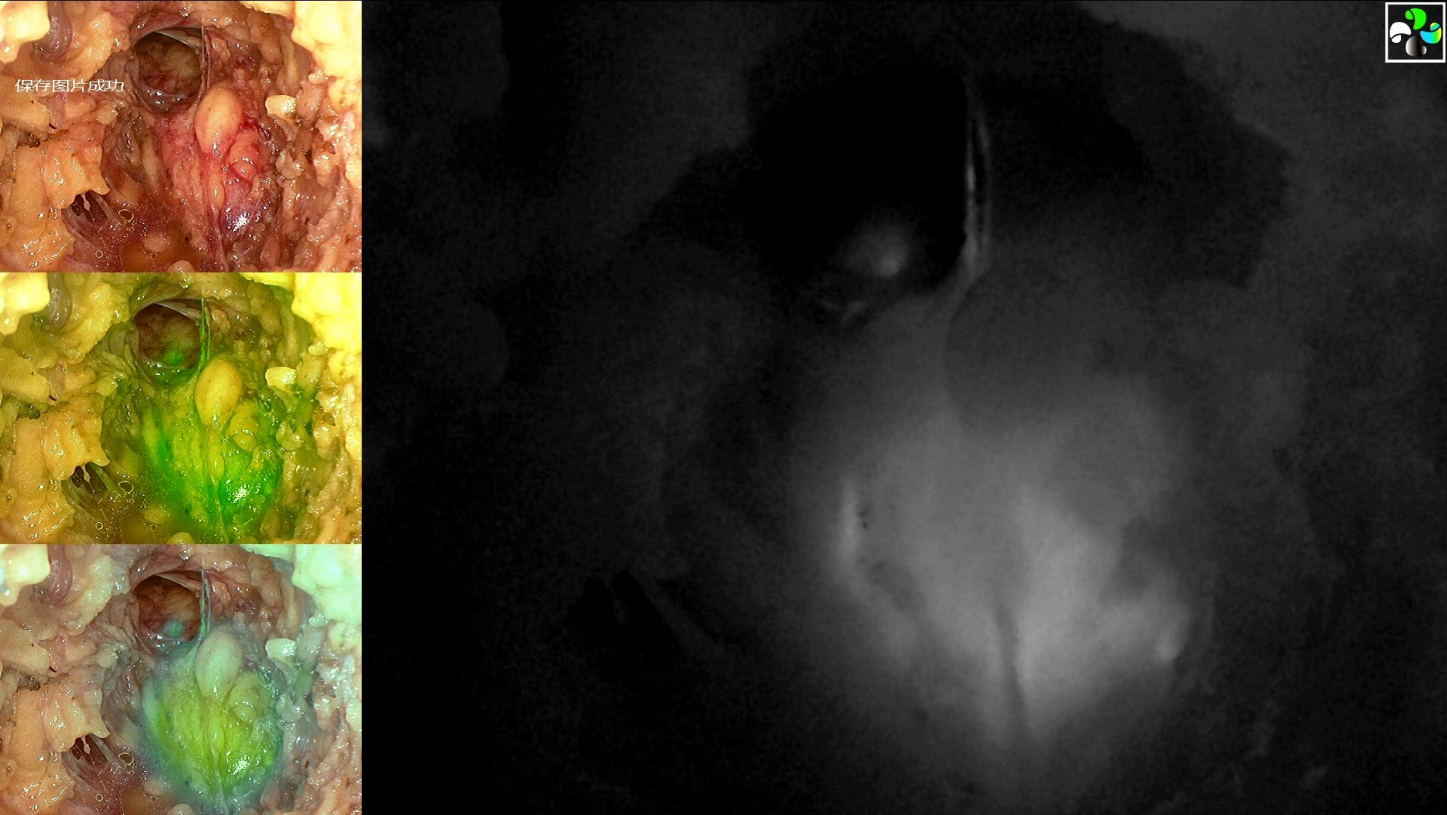
Example of parallel display mode. The near-infrared fluorescence endoscopy can easily detect the fluorescence of the indocyanine green in the sentinel lymph nodes and the lymphatic vessels connected to them

**Fig. 5:**
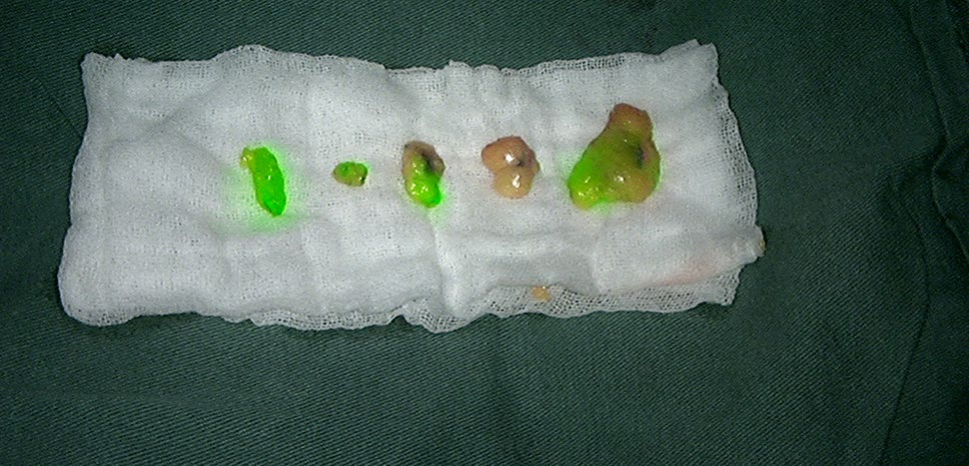
The left and right lymph nodes were stained with both indocyanine green (fluorescence) and carbon nanoparticles (black stain). The middle lymph node was only stained with carbon nanoparticles (Color figure online)

A small single-port incision about 2.5 cm in length was created with the single-port insufflation kit (HTKD-Hang T Port, China) (Fig. 2) at the axillary midline flush with the nipple and filled with CO_2_ gas. The pressure was maintained at 8 mmHg (1 mmHg = 0. 133 kPa), and the gas flow rate was kept at 8 L/min. This established adequate working space for the operation. Then, endoscopic surgical instruments and the near-infrared fluorescence endoscopy of FloNaviTM Endoscopic Fluorescence Imaging System (Optomedic Technique Inc., Guangdong, China) (Fig. 3) were implanted through the single-port insufflation kit. The endoscopic fluorescence imaging system emits an excitation light at 760 nm, which produces the fluorescence of ICG that is displayed by computer processing. The near-infrared fluorescence endoscopy also magnifies the area to easily detect the fluorescence of the ICG in the SLNs and the lymphatic vessels connected to it (Fig. 4). The lymphatic vessels surrounding the SLNs were clipped, and the SLNs visible with fluorescent lymph nodes (ICG +) and/or black-stained lymph nodes (CN +) were removed (Fig. 5).

The SLN specimens were removed with a specimen bag and sent for intraoperative frozen pathology. Patients who did not conform to the Z0011 criteria continued to receive endoscopic axillary lymph node dissection through the single-port incision. On the contrary, if a patient met the Z0011 criteria, then the surgical field was washed with physiological saline, and a silicone drainage tube was placed to connect the negative pressure suction. The drainage tube was removed three days after operation. Patients receiving single-port endoscopic subcutaneous mastectomy, breast-conserving surgery, or implanted breast reconstruction were performed through the same single-port incision to complete the follow-up single-port endoscopic surgery. Specific procedures and techniques of single-port endoscopic breast surgery were described previously [6].

#### Group 2: SPE-SLNB with CNs

CNs (0.5 mL) were injected into the outer and lower edge of the areola. The placement of the patient’s posture, liposuction, and the establishment of surgical space were the same as in the SPE-SLNB/ICG/CN group. The endoscopic instruments and laparoscope were implanted through the single-port insufflation kit. Then, we separated the fibrous connective tissue in front of the lens, identified the black-stained SLN and its connected lymphatic vessels, clipped the lymphatic vessels around the SLN, and removed all lymph nodes stained with CNs. The extraction, treatment, and other surgical procedures of the specimens were the same as in the SPE-SLNB/ICG/CN group.

#### Group 3: C-SLNB combined with ICG and CNs

Intradermal injection of 0.3 mg/mL of ICG and 0.2 mL of CNs were injected at the outer and lower margins of the areola, respectively. Then, ICG fluorescence was excited and detected by an in vitro hand-held fluorescence detector (Optomedic Technique Inc., Guangdong, China), and lymphatic drainage was tracked in real time on the monitor. The incision was made 1 cm away from the disappearance of the fluorescence. The fluorescent lymph nodes (ICG +) and/or black-stained lymph nodes (CN +) were detected under direct vision, and the C-SLNB was completed. If the fluorescent lymphography was undetected, then we made a routine incision in the axilla of the patient. When the skin and subdermal fat were incised, the black-stained lymph nodes were resected. Once the SLN specimens were removed, they were sent for intraoperative frozen pathology. The removal of specimens and the treatment of the axilla were the same as the SPE-SLNB/ICG/CN group. Finally, the breast surgery was performed through traditional open surgery.

For all groups, if the SLN metastasis fulfilled the Z0011 criteria, then an axillary lymph node dissection would not be performed.

### Evaluation of SLNs and arm function

The detection rate of SLNs, mean number of SLNs, and the physical function of the upper limbs were evaluated to determine the efficacy and safety of the procedures. SLNs detection rate = number of sentinel lymph nodes detected/ total number of each group. In order to evaluate the physical function of the upper limbs, clinicians performed sensory evaluation at one month and 6 months after surgery. The patients were contacted by telephone for follow-up. The upper arm pain score was measured by the visual analogue scale. If the pain score = 0, then it was reported as no pain; if the pain score = 1–3, then it was reported as mild pain; and if the pain score ≥ 4, then it was reported as moderate to severe pain. The degree of sensory loss at the upper arm was also measured by the visual analogue scale. The scale ranged from 0 (no change in sensation) to 10 (complete loss of sensation).

### Statistical analysis

Variables between the three surgical techniques were compared using the chi-squared test and independent sample *t*-test. All statistical analyses were performed using IBM SPSS Statistics ver. 26.0 (IBM Co., Armonk, NY, USA) software, and a *p*-value < 0.05 represented statistical significance.

## Results

### Patient and tumor characteristics

Detailed information about patient characteristics and tumors is shown in Table 1. There were no differences in mean age, body mass index, histological classification, menopause status, or tumor localization between the patients in the three groups. There were statistical differences in tumor size and laterality.

**Table 1:** Patient and tumor characteristics

Characteristic	Group 1 (*n* = 20)	Group 2 (*n* = 20)	Group 3 (*n* = 20)	*p*-value
Age (yr)				
≤ 50	6	10	12	0.153
> 50	14	10	8	
Body mass index (kg/m2)				
≤ 18.4	0	1	0	0.540
18.5–23.9	12	11	10	
24.0–27.9	8	7	9	
≥ 28.0	0	1	1	
Histologic type				
Invasive ductal	17	18	17	0.729
Invasive lobular	1	1	0	
Ductal carcinoma in situ	2	1	3	
Other types	0	0	0	
Tumor size (cm)				
< 2.0	15	17	7	0.002
2–5	5	3	13	
Menopause				
Pre	5	6	10	0.215
Post	15	14	10	
Laterality				
Left	12	5	3	0.007
Right	8	15	17	
Tumor localization				
Upper outer	11	7	9	0.283
Lower outer	6	8	8	
Upper medial	2	0	2	
Lower medial	1	2	1	
Central	0	3	0	
Estrogen receptor status				
Positive	5	3	1	0.065
Negative	15	17	19	

Group 1: single-port endoscopic-sentinel lymph node biopsy combined with indocyanine green and carbon nanoparticles; Group 2: single-port endoscopic-sentinel lymph node biopsy with carbon nanoparticles only; Group 3: conventional sentinel lymph node biopsy with indocyanine green and carbon nanoparticles

### SLN detection

The SLN detection rates for Group 1、Group 2 and Group 3 were 100%, 100%, and 95%, respectively. SLNs were detected successfully in Group 2 and the Group 2 The SLN in one patient in Group 3 was not detected. The detection rates of the Group 1 and Group 3 and of Group 2 and Group 3 were compared separately, and there were no statistical differences (*p* > 0.05).

Fluorescent lymphography was visible in the Group 1 and Group 3. In Group 1, the fluorescent lymphangiography and fluorescent SLN was detected in 20 patients. Transcutaneous fluorescent lymphography or black-stained lymph nodes were visible in 19 patients in Group 3.

In Group 1, Group 2 and Group 3, the total number of SLNs detected was 97 (68 were ICG + /CN + , 27 were ICG + /CN-, and 2 were ICG-/ CN +), 65 (CN +), and 98 (68 were ICG + /CN + , 30 were ICG + /CN-, and 0 were ICG-/ CN +), respectively. In addition, there was a significant difference in the mean number of SLNs between Group 1 (4.85 ± 2.28, range: 1–9) and Group 2 (3.25 ± 1.77, range: 0–6) (p < 0.05). There was a significant difference in the mean number of SLNs between the Group 2 and Group 3 (4.90 ± 2.27, range: 1–9) (p < 0.05). There was no statistical difference between Group 1 and Group 3.

The total number, mean number, detection rate of SLNs and the number of metastatic SLNs is shown in Table 2.

**Table 2:** The total number, mean number, detection rate of SLNs and the number of metastatic SLNs

	Group1	Group2	Group3
Total number of SLNs	97	65	98
Mean number of SLNs	4.85 ± 2.28	3.25 ± 1.77	4.90 ± 2.27
Number of metastatic SLNs	7	2	6
SLNs detection rate	100%	100%	95%

Group 1: single-port endoscopic-sentinel lymph node biopsy combined with indocyanine green and carbon nanoparticles; Group 2: single-port endoscopic-sentinel lymph node biopsy with carbon nanoparticles only; Group 3: conventional sentinel lymph node biopsy with indocyanine green and carbon nanoparticles

### Metastasis rate of SLNs

In Group 1, three tumor-positive patients were detected with seven metastatic SLNs. In Group 2, there was one positive patient with two metastatic SLNs. In Group 3, there were two positive patients with six metastatic SLNs (three were ICG + /CN + , three were ICG + /CN-, and zero were ICG-/CN +).

### The physical function of the upper limbs

The average pain scores of Group 1、Group 2 and Group 3 at one month after surgery were 2.65 ± 0.93, 2.35 ± 1.03, and 3.20 ± 0.70, respectively. The average pain scores of Group 1、Group 2 and Group 3 at six months after surgery were 0.95 ± 0.69, 1.15 ± 0.81, 1.50 ± 0.76, respectively. The average sensory loss scores of Group 1、Group 2 and Group 3 at one month after surgery were 5.40 ± 0.88, 5.35 ± 1.08, and 5.50 ± 0.89, respectively. The average sensory loss scores of Group 1、Group 2 and Group 3 at six months after surgery were 4.70 ± 0.92, 4.40 ± 1.35, and 4.65 ± 1.09, respectively. There were no statistical differences between the three groups in average pain scores and average sensory loss scores. The pain and sensory loss scores at one month and six months after surgery is shown in Table 3.

**Table 3:** Pain and sensory loss scores at one month and six months after surgery

	Group1	Group2	Group3	*P* value#
Average pain score at one month	2.65 ± 0.93	2.35 ± 1.03	3.20 ± 0.70	0.081
Average pain score at six months	0.95 ± 0.69	1.15 ± 0.81	1.50 ± 0.76	0.075
Average sensory loss score at one month	5.40 ± 0.88	5.35 ± 1.08	5.50 ± 0.89	0.881
Average sensory loss score at six months	4.70 ± 0.92	4.40 ± 1.35	4.65 ± 1.09	0.672

^#^The ANOVA test was used to evaluate differences between the three groups. Group 1: single-port endoscopic-sentinel lymph node biopsy combined with indocyanine green and carbon nanoparticles; Group 2: single-port endoscopic-sentinel lymph node biopsy with carbon nanoparticles only; Group 3: conventional sentinel lymph node biopsy with indocyanine green and carbon nanoparticles

## Discussion

SLNs are the first lymph nodes that cancer cells are most likely to spread to from the primary tumor. The accurate and complete visualization of the lymphatic vessels and SLNs is crucial for detecting metastasis and accurately staging cancer. ICG-enhanced fluorescence was introduced to improve the detection of SLNs in early breast cancer during conventional open surgery and was found to be safe and effective [11, 12]. However, in C-SLNB the location of the lymph node is judged by the position of the disappearance of the lymphatic vessel and cannot be accurately detected before creating an incision. Therefore, our center uses SPE-SLNB combined with an endoscopic fluorescence imaging system that magnifies and directly excites the ICG to overcome this disadvantage. However, few studies have shown if SPE-SLNB has similar detection rates and is as safe as C-SLNB. Without this kind of study, when we perform endoscopic breast surgery, we need to perform the second incision for open C-SLNB, which could increase the trauma and affect the aesthetic outcome. In this study, our center tested a novel method of using ICG as a tracer while performing SPE-SLNB in order to accomplish SLNB with the same single-port incision used for the endoscopic breast surgery. Therefore, we could effectively perform two procedures using a single-port.

There was no significant difference in the SLN detection rates between the three groups. This result indicates that SPE-SLNB combined with ICG and CNs is just as reliable at detecting SLNs as C-SLNB. It should be noted that in the C-SLNB/ICG/CN group, detection of the SLNs through the skin failed in one patient. This is due to the limitation of approximating the position of the SLN by the disappearance of the fluorescent lymphatic vessels. Kitai et al. [13] reported a similar observation in which the fluorescence of ICG in one patient out of eighteen who received C-SLNB was not detected. Similarly, Guo et al. [14], who used ICG combined with a methylene blue tracer, found that fluorescent SLNs through the skin was not detected in 16/200 cases of C-SLNB. In conventional surgery, only the fluorescence excited by the superficial lymphatic vessels could be detected after the injection of ICG. However, the fluorescence of SLNs that are further from the body surface cannot be directly detected by conventional methods before creating an incision. For this reason, the detection rate of the C-SLNB/ICG/CN group was lower than that of the SPE-SLNB/ICG/CN group and the SPE-SLNB/CN group.

To overcome the challenge of detecting SLNs further from the body surface, our center established the use of ICG-enhanced fluorescence imaging with a near-infrared fluorescence endoscope while performing SPE-SLNB. The near-infrared fluorescence endoscope placed deep in the axillary enlarges the SLNs and directly visualizes the SLNs that emit strong fluorescence. This avoids the inability of detecting SLNs hidden deep within the skin and subcutaneous tissue during C-SLNB. Moreover, when performing other single-port endoscopic breast surgery, such as single-port endoscopic breast-conserving surgery, single-port endoscopic subcutaneous mastectomy, and single-port endoscopic breast reconstruction, it is advantageous to perform the SLNB through the single-port incision. By combining ICG and SPE-SLNB, we are able to reliably accomplish the axillary lymph node staging with the same single-port incision.

The mean number of SLNs detected in Group 1and Group 3 was significantly more than that of Group 2 (*P* < 0.05), indicating that ICG confers an advantage on the detection of SLNs. The mean number of SLNs detected between Group 1 and Group 3 was not statistically significant (*P* = 0.944). This result shows that SPE-SLNB can detect as many SLNs as C-SLNB, indicating the reliability of this new technique. According to previous studies [15–17], increasing the number of SLNs removed will lead to improved accuracy and a decreased false-negative rate. When the detection number of SLNs reaches 3–4, the accuracy rate is stable at 95%, and the false-negative rate is below 5%.

When comparing Group 1 with Group 2, the results suggested that the use of ICG significantly increased the mean number of SLNs detected. This is likely due to the novel use of the near-infrared fluorescence endoscope. The use of this tool allows the surgeon to directly visualize the SLN that is emitting the fluorescence. It is also important to note that ICG has a stronger affinity for the lymphatic system than CNs due to the molecular structure and diameter of ICG [18]. Another reason may be that the detection of CNs is via the naked eye, and interference caused by electric knife eschar and intraoperative bleeding would affect identification of CN-traced SLNs. In contrast, ICG is excited via the endoscopic fluorescence imaging system, which allows for the fluorescence to be easily detected. Moreover, in the SPE-SLNB/ICG/CN group, five of the seven metastatic SLNs were ICG + /CN + , and zero were ICG-/CN + . In the C-SLNB/ICG/CN group, three of the six metastatic SLNs were ICG + /CN + , and zero were ICG-/CN + . This indicates that ICG is a reliable tracer for metastatic SLNs. Importantly, two metastatic SLNs were ICG + /CN- in Group 1, and three metastatic SLNs were ICG + /CN- in Group 3. If ICG had not detected these metastatic SLNs, then the patients would have received false-negative results.

Patients who receive axillary surgery may suffer from nerve injury and shoulder dysfunction as a result [19]. We observed that there were no statistically significant differences in pain or sensory loss between the three surgical groups at one month after surgery and six months after surgery. Another study reported that patients who received endoscopic SLNB had less anesthesia and upper limb pain after surgery than patients who received open C-SLNB (20). This discrepancy may be due to the small sample size of this study. However, this observation shows that SPE-SLNB and C-SLNB affect the upper limb in a similar way and thus displays the safety of this surgical technique.

Because this study was not a randomized study, some of the patient characteristics, such as the tumor size and laterality (which do not affect SLNB), were different between the three groups. A randomized controlled trial with a larger sample size is needed to further confirm the benefits of SPE-SLNB combined with ICG and CN observed in this study.

In conclusions, the novel combination of the single-port, the use of ICG, and the introduction of the near-infrared fluorescence endoscope can achieve a satisfactory SLN detection rate while performing other endoscopic breast surgeries. We observed that the application of SPE-SLNB combined with ICG and CNs is as safe and efficacious as C-SLNB with the added benefit of reducing the number of missed SLNs and the subsequent false-negative rate. The most important advantage of SPE-SLNB is that two procedures can be effectively performed through a single-port in the axilla.

## Abbreviations

ICG: Indocyanine green
SLNB: Sentinel lymph node biopsy
SPE-SLNB: Single-port endoscopic-sentinel lymph node biopsy
CN: Carbon nanoparticle
C-SLNB: Conventional sentinel lymph node biopsy
SLN: Sentinel lymph node

## Declarations

### Reporting checklist

The authors have completed the STARD reporting checklist.
